# New measure of functional tooth loss for successful Oral ageing: a cross-sectional study

**DOI:** 10.1186/s12877-023-04570-1

**Published:** 2023-12-15

**Authors:** Yiting Cheng, Li Cheng, Fangzhi Zhu, Yong Xiang, Shaoying Duan, Jingjing Luo, Lei Lei, He Cai, Tao Hu

**Affiliations:** State Key Laboratory of Oral Diseases & National Center for Stomatology & National Clinical Research Center for Oral Diseases, West China Hospital of Stomatology, Sichuan University, 14#, 3rd section, Renmin South Road, Chengdu, 610041 Sichuan China

**Keywords:** Successful ageing, Geriatric dentistry, Quality of life, Oral health, Functional health status, Risk factor, Epidemiology

## Abstract

**Background:**

This cross-sectional study evaluated the impacts of functional tooth loss on oral health-related quality of life (OHRQoL) among elderly people compared with the impacts of several common indicators of oral health. Additionally, the cut-off of functional tooth loss needed for a better OHRQoL was investigated to establish a new measure for successful oral ageing.

**Methods:**

Data from people aged 65–74 were extracted from the Fourth National Oral Health Survey in Sichuan, China. Functional tooth loss was defined as both natural tooth loss and nonfunctional teeth, such as third molars, residual roots, and removable dentures. The cut-offs of tooth loss were first identified as 12, based on the previous definition of functional dentition (≥20 natural teeth except the third molars), and 14, 16, or 18 for further investigation. OHRQoL was evaluated by the standardized Geriatric Oral Health Assessment Index (sGOHAI) score. Logistic regression was performed to estimate the impacts on OHRQoL. Additionally, subgroup analyses were conducted using the stratified chi-square test to explore the effect of functional tooth loss at each position.

**Results:**

The mean GOHAI score of the 744 participants was 48.25 ± 7.62. Elderly people who had lost ≤12 functional teeth had greater odds of reporting a higher sGOHAI score than those who had lost more functional teeth (odds ratio (OR) 1.49, 95% confidence interval (CI) 1.05–2.11). No significant difference in the sGOHAI score was detected between people who had lost 13–16 functional teeth and those who had lost ≤12 functional teeth (0.61, 0.35–1.07). The loss of second premolars and first and second molars had great impacts on the sGOHAI score when ≤12 or ≤ 16 functional teeth had been lost.

**Conclusions:**

Compared with natural tooth loss, functional dentition and occluding pairs, functional tooth loss can be a better indicator of OHRQoL in the elderly population. Sixteen remaining functional teeth seem to be sufficient to maintain good OHRQoL and successful oral ageing despite that number being previously acknowledged as ≥20 teeth.

## Background

With the global demographic transition, including an increase in life expectancy and a growth in the proportion of older people, there is an urgent need for geriatric oral health care [1–3]. The World Health Organization (WHO) states that population ageing can result in difficulties in the management and maintenance of acceptable oral health and necessary oral functions [1]. Consequences of poor oral health among older adults can include pain and suffering, aesthetic concerns, psychosocial distress, and adverse impacts on the quality of life in this specific vulnerable population [4–7], which can hardly achieve successful oral ageing, the process of developing and maintaining oral function that enables well-being in old age [8]. Therefore, oral health-related quality of life (OHRQoL), which indicates the self-perceived impacts of oral health on physical and psychosocial well-being [9], has generally been considered an important patient-centred parameter to comprehensively evaluate an individual’s oral health status, particularly among older persons [10–12].

Tooth loss reflects the complex outcome of oral diseases and the individual’s history of dental treatment over the life course [4, 13] and is considered a common indicator of OHRQoL [14–16]. However, the results of the literature are still inconsistent regarding the relationship between tooth loss/retention and OHRQoL [1, 17]. Some research has shown that the number of natural teeth might have a positive association with OHRQoL [18], and the cut-off of the number of teeth indicating successful oral ageing (which is also called functional dentition by the WHO) associated with a higher OHRQoL varied between 20 and 21 [13, 19]. However, some studies reported that there was no significant association between the number of natural teeth and OHRQoL [1, 18]. Instead, some researchers have suggested that the number of occluding pairs might have more potential to identify OHRQoL [20, 21], but notably, it might take more time and effort to determine and record the occlusal relationship than to simply count the number of teeth.

Teeth that function well in mastication, including sound natural teeth, teeth with early-stage caries, teeth with enamel, teeth with dentin decay, and filled teeth, are defined as functional teeth, while teeth with severe or pulpal decay and stump teeth, third molars and residual roots are considered nonfunctional teeth [22, 23]. Removable dentures are regarded as nonfunctional due to their controversial effect on oral function [24]. Therefore, we further defined “functional tooth loss” as follows: natural and nonfunctional tooth loss, such as the third molars, residual roots, and the use of removable dentures. To our knowledge, no studies have confirmed the association between functional tooth loss and OHRQoL.

Thus, this study aimed to determine the relationship between functional tooth loss and OHRQoL in older people compared with the relationships between OHRQoL and several common indicators (i.e., natural tooth retention, functional dentition, and occluding pairs) of oral health. A significant association was assumed to be found between functional tooth loss and OHRQoL in the older population (hypothesis). Additionally, the cut-off of the number of missing functional teeth (functional tooth loss) associated with a high OHRQoL was investigated to establish a new measure for successful oral ageing, providing clinicians and clinical researchers with a new approach for clinical decision-making and support for the formulation of relevant oral health care policies.

## Methods

The present observational study followed the Strengthening the Reporting of Observational Studies in Epidemiology (STROBE) statement, and all relevant information was reported according to the STROBE checklist for cross-sectional studies.

### Study design

As a part of the Fourth National Oral Health Survey of China, this cross-sectional study was completed in Sichuan in 2016, after the validation of the pretest finished in the same target population in August 2015 [17, 25, 26]. Ethical approval was granted by a local stomatological ethics committee. Data from participants aged 65–74 years were used in this study.

### Survey sampling

A four-stage stratified random cluster sampling was conducted using the probability proportional to size (PPS) method [25]. Six areas, including three districts and three counties, were randomly selected in the first two stages. Three communities were then selected in each respective area, and participants were randomly selected by a quota sampling method in the fourth stage [25]. Individuals with serious physical or psychological illness or disadvantages and those who were unable or unwilling to complete the survey were excluded [25, 27].

### Subjects

The target population was residents aged 65–74 years, and the minimum required sample size of 696 was calculated based on the following formula [25]:

$$n= deff\frac{\mu^2p\left(1-p\right)}{\varepsilon^2}/\left(1- non- response\ rate\right)$$

where *deff* as the design efficiency was 2.5; *p* as the prevalence of caries in this population of older people in the Third National Oral Health Survey was 86.0%; the nonresponse rate was 15%; *μ* as the level of confidence was 1.96; and ε as the margin of error was 10%.

### Clinical examination

According to the fifth version of the World Health Organization Oral Health Survey-Basic Methods [28], participants received an oral clinical examination executed by three licenced dentists (LC, JL, LL) and recorded by three trained individuals (HC, FZ, SD). All examiners were trained by the standard examiner and passed the consistency check before this study. Interobserver variability was analysed, and the mean kappa values were both > 0.85.

**Fig. 1:**
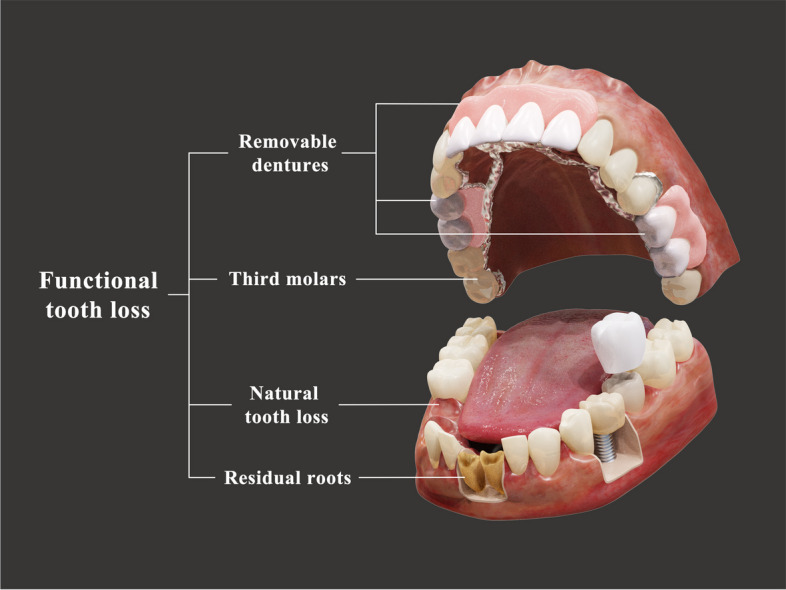
Definition of functional tooth loss. Functional tooth loss was defined to include both natural tooth loss and nonfunctional teeth, such as residual roots, third molars, and removable dentures

The standardized oral examination focused on dentition status, including tooth loss, coronal and root caries, and denture conditions. Teeth missing for any reason were classified as natural tooth loss. A number of remaining natural teeth (except the third molars, which are usually nonfunctional) ≥20 was considered functional dentition [22, 23, 29]. Tooth pairs with occlusal contact, according to the normal tooth position in centric occlusion, were regarded as occluding pairs [30]. Functional tooth loss was defined to include both natural tooth loss and nonfunctional teeth (those remaining but not achieving oral function, i.e., residual roots, third molars, etc.) (Fig. 1). Removable dentures were also included as functional tooth loss, as their effects on oral function are controversial [31, 32]. Fixed prostheses and dental implants were considered remaining functional teeth because recent studies have proven their positive effects on OHRQoL [33, 34]. The number 12 was identified as the cut-off of natural tooth loss, which equalled 20 remaining natural teeth [13, 18, 19]. Similarly, the cut-off of functional tooth loss was also identified to be 12, which equalled 20 functional remaining teeth. To explore the minimum number of functional teeth needed for better OHRQoL, 14 was also taken as the cut-off value, which divided the number of functional tooth losses into three groups: > 14, 13–14, and ≤ 12, as well as 16 and 18. Additionally, participants were identified into six classes (complete dentition, type I, type II, type III, type IV, edentulous) by two independent, experienced dentists (LC, HC) according to the Kennedy classification, and any disagreements were resolved through discussion with a third assessor (TH).

### Questionnaire

Participants’ basic information, including demographic variables (age, sex, educational status, and household annual income), health behaviours including smoking and drinking habits, medical history, and self-rated oral health status, was extracted from a self-report questionnaire, and all records were verified before entry. The participants’ educational status was classified into two levels: “low” (“junior high school” or below) and “high” (“high school” or above). Household annual income per capita was categorized into three categories (<10, 10–20, > 20 thousand) [35]. Smoking and drinking habits were dichotomized as “yes” (“past or current smoking/drinking”) or “no” (“never smoking/drinking”). Medical history was evaluated as “yes” or “no” according to the presence of any systemic diseases. Self-rated oral health status was evaluated by a 1–5 Likert scale: “very poor”, “poor”, “fair”, “good”, and “very good”. The variables above and tooth loss were considered explanatory variables for the analyses with OHRQoL.

OHRQoL was measured in the questionnaire using the Chinese version of the 12-item Geriatric Oral Health Assessment Index (GOHAI), which evaluated the impacts of oral conditions on daily activities, such as physical function, pain and discomfort, and psychosocial function [36]. Responses were made on a 5-point Likert scale (1 = “always”, 2 = “often”, 3 = “sometimes”, 4 = “seldom”, and 5 = “never”). The total scores for the GOHAI were summed by the ordinal value of each question, ranging from 12 to 60, where a higher GOHAI score indicated a better OHRQoL. To obtain a clearer distribution of GOHAI scores, we applied the following formulas to convert a raw score into a standardized score [37]:

$$\textrm{Transformed}\ \textrm{Score}=\left[\frac{\left(\textrm{Actual}\ \textrm{raw}\ \textrm{score}-\textrm{lowest}\ \textrm{possible}\ \textrm{raw}\ \textrm{score}\right)}{\textrm{Possible}\ \textrm{raw}\ \textrm{score}\ \textrm{range}\ \left(\textrm{highest}\ \textrm{score}-\textrm{lowest}\ \textrm{score}\right)}\right]\times 100$$

$$\textrm{Standardized}\ \textrm{Score}=\left[\frac{\left(\textrm{Transformed}\ \textrm{score}-\textrm{the}\ \textrm{mean}\ \textrm{score}\ \textrm{of}\ \textrm{all}\ \textrm{samples}\right)}{\textrm{The}\ \textrm{standard}\ \textrm{deviation}\ \textrm{of}\ \textrm{all}\ \textrm{samples}}\right]\times 10+50$$

The transformed GOHAI scores in all categories ranged from 0 to 10, and the mean standardized GOHAI (sGOHAI) score was 50, which was then divided into two categories: “low” (sGOHAI score ≤ 50) or “high” (sGOHAI score > 50).

### Statistical analysis

All questionnaires and examination reports were double-entered by two trained dentists (YC, YX). Categorical and continuous variables are presented as prevalences and proportions or means±standard deviations (SDs). The frequency of functional tooth loss at each tooth position is shown in heatmaps, demonstrated by a commonly used thermogram and heatmaps shown in dentitions. The frequency of the Kennedy classifications is shown in a circular graph. Binary logistic regression was performed to calculate unadjusted and adjusted (sex, age, educational status, household annual income level, smoking and drinking habits, medical history, and self-rated oral health) odds ratios (ORs) and 95% confidence intervals (95% CIs), analysing the relationships of functional tooth loss, natural tooth loss, functional dentition, and occluding pairs with the total sGOHAI score and 12 sGOHAI item scores. Binary logistic regression was also used to evaluate the relationships among three groups of functional tooth loss with different cut-offs (12, 14, 16, and 18) and sGOHAI scores. Further analyses were conducted to explore the associations between the positions of the lost teeth and the sGOHAI score in different subgroups of people with ≤12, > 12, ≤16 or > 16 missing functional teeth using a stratified chi-square test. SPSS Statistics V.26.0 (IBM Corp., Armonk, NY) was used for data analysis and statistics.

## Results

A total of 744 participants (48.7% males) were included in this study. All participants completed the clinical examination and the questionnaire. The mean age of the study population was 68.48 ± 2.76 years, and 86.2% of them had an educational level of no higher than junior high school. More than half of the subjects (61.8%) had been previously diagnosed with at least one systemic ailment. The demographic characteristics of the participants are reported in Table 1.

**Table 1:** Demographic characteristics of the participants (*n* = 744)

Variables	All Participants	Participants with sGOHAI score > 50	Participants with sGOHAI score ≤ 50
	Count (%)/Mean ± SD	Count (%)/Mean ± SD	Count (%)/Mean ± SD
**Sex**			
Male	362 (48.7%)	209 (51.2%)	153 (45.5%)
Female	382 (51.3%)	199 (48.8%)	183 (54.5%)
**Age, years**	68.48 ± 2.76	68.59 ± 2.82	68.36 ± 2.69
**Education level**			
Low	641 (86.2%)	343 (84.1%)	298 (88.7%)
High	103 (13.8%)	65 (15.9%)	38 (11.3%)
**Level of household annual income, per capita (thousand)**			
<10	245 (32.9%)	120 (29.4%)	125 (37.2%)
10–20	256 (34.4%)	150 (36.8%)	106 (31.5%)
> 20	243 (32.7%)	138 (33.8%)	105 (31.3%)
**Smoking habits**			
Yes	260 (34.9%)	161 (39.5%)	99 (29.5%)
No	484 (65.1%)	247 (60.5%)	237 (70.5%)
**Drinking habits**			
Yes	359 (48.3%)	211 (51.7%)	148 (56.0%)
No	385 (51.7%)	197 (48.3%)	188 (44.0%)
**Medical history**			
Yes	460 (61.8%)	231 (56.6%)	229 (68.2)
No	284 (38.2%)	177 (43.4%)	107 (31.8)
**Self-rated oral health**			
Poor or very poor	261 (35.1%)	82 (20.1%)	179 (53.3%)
Fair	296 (39.8%)	177 (43.4%)	119 (35.4%)
Good or very good	187 (25.1%)	149 (36.5%)	38 (11.3%)
**Number of functional tooth loss**			
> 12	248 (33.3%)	112 (27.5%)	136 (40.5%)
≤ 12	496 (66.7%)	296 (72.5%)	200 (59.5%)
**Number of functional tooth loss**			
> 14	202 (27.2%)	85 (20.8%)	117 (34.8%)
13–14	46 (6.2%)	27 (6.6%)	19 (5.7%)
≤ 12	496 (66.7%)	296 (72.5%)	200 (59.5%)
**Number of functional tooth loss**			
> 16	172 (23.1%)	72 (17.6%)	100 (29.8%)
13–16	76 (10.2%)	40 (9.8%)	36 (10.7%)
≤ 12	496 (66.7%)	296 (72.5%)	200 (59.5%)
**Number of functional tooth loss**			
> 18	142 (19.1%)	62 (15.2%)	80 (23.8%)
13–18	106 (14.2%)	50 (12.3%)	56 (16.7%)
≤ 12	496 (66.7%)	296 (72.5%)	200 (59.5%)
**Number of tooth loss**			
> 12	150 (20.2%)	151 (37.0%)	169 (50.3%)
≤ 12	594 (79.8%)	257 (63.0%)	167 (49.7%)
**Functional dentition**			
Yes	161 (21.6%)	74 (18.1%)	87 (25.9%)
No	583 (78.4%)	334 (81.9%)	249 (74.1%)
**Number of occluding pairs**	9.18 ± 4.75	9.65 ± 4.67	8.61 ± 4.79
**GOHAI score**	48.25 ± 7.62	53.79 ± 3.31	41.53 ± 5.73
**sGOHAI score**	50.00 ± 10.00	57.26 ± 4.34	41.18 ± 7.52

*GOHAI score* General Oral Health Assessment Index score, *sGOHAI score* standardized GOHAI by the mean score, *SD* standard deviation

The mean GOHAI score was 48.25 ± 7.62, with a median score of 49, which was converted to 57.26 ± 4.34 after standardization. A total of 54.8% of the elderly participants had a better OHRQoL (sGOHAI score > 50) than the others. For responses to each item, almost half of the subjects reported problems with food selection and biting/chewing, while nearly one in every three older people indicated sensitive gums or showed worry or concern about their oral health problems (Table 2).

**Table 2:** GOHAI items and frequency distribution of the responses

Items		Frequency (%)				
		1 Always	2 Often	3 Sometimes	4 Seldom	5 Never
**Physical function**						
**1**	Limit the kind of food	14.2%	31.5%	17.9%	11.3%	25.1%
**2**	Trouble biting/chewing	10.5%	31.3%	19.2%	11.2%	27.8%
**3**	Trouble swallowing	1.1%	4.7%	11.2%	8.7%	74.3%
**4**	Unable to speak clearly	1.5%	3.1%	7.7%	6.5%	81.3%
**Pain and discomfort**						
**5**	Discomfort when eating	3.8%	13.7%	18.5%	11.7%	52.3%
**8**	Medications for pain	2.4%	9.9%	20.4%	20.4%	46.8%
**12**	Sensitive gums	11.0%	22.7%	19.4%	8.5%	38.4%
**Psychosocial impacts**						
**6**	Limit contact with others	0.7%	3.1%	6.3%	7.1%	82.8%
**7**	Unhappy with appearance	2.2%	9.4%	11.7%	13.3%	63.4%
**9**	Worried or concerned	4.2%	23.7%	16.8%	20.2%	35.2%
**10**	Nervous, self-conscious	1.7%	4.6%	7.3%	9.5%	76.9%
**11**	Uncomfortable eating in front of others	1.5%	5.0%	8.5%	10.2%	74.9%

*GOHAI score* General Oral Health Assessment Index score

**Fig. 2:**
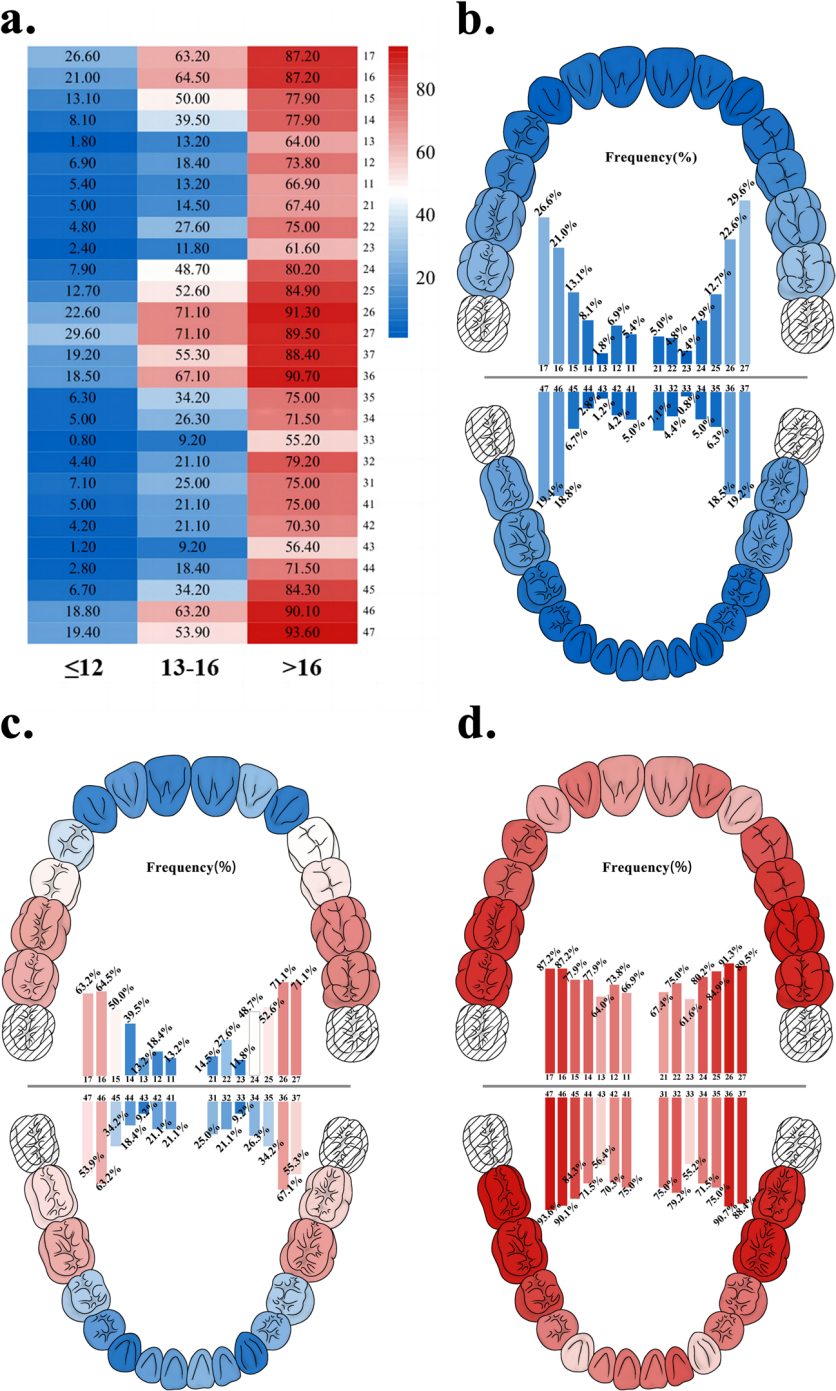
The frequency of functional tooth loss for each tooth position. The dentitions show the frequency of functional tooth loss in each tooth position among elderly people with functional tooth loss ≤12, 13–16, and > 16, from 0% in blue to 100% in red. **a** The heatmap summarizing the frequency of functional tooth loss in each position in three groups; and **b**-**d** the frequency of functional tooth loss in each position among people who lost ≤12, 13–16, and > 16 functional teeth, where the upper and lower panel shows the frequency of functional tooth loss in maxillary and mandibular jaws, and the middle panel shows the bar chart for the frequency of functional tooth loss in each tooth position

Regarding oral health status, 7.9% of the participants were edentulous, 71.9% had ≥20 natural teeth, and 20.2% had lost > 12 teeth (<20 teeth remained). The mean numbers of functional tooth loss, natural tooth loss and occluding pairs were 10.24 ± 9.18, 8.12 ± 8.35 and 9.18 ± 4.75, respectively. The frequency of molars lost was much higher than that of premolars, followed by incisors and canines. Maxillary teeth were more likely to be lost than mandibular teeth, but there was no significant difference between the left and right sides (Fig. 2).

**Fig. 3:**
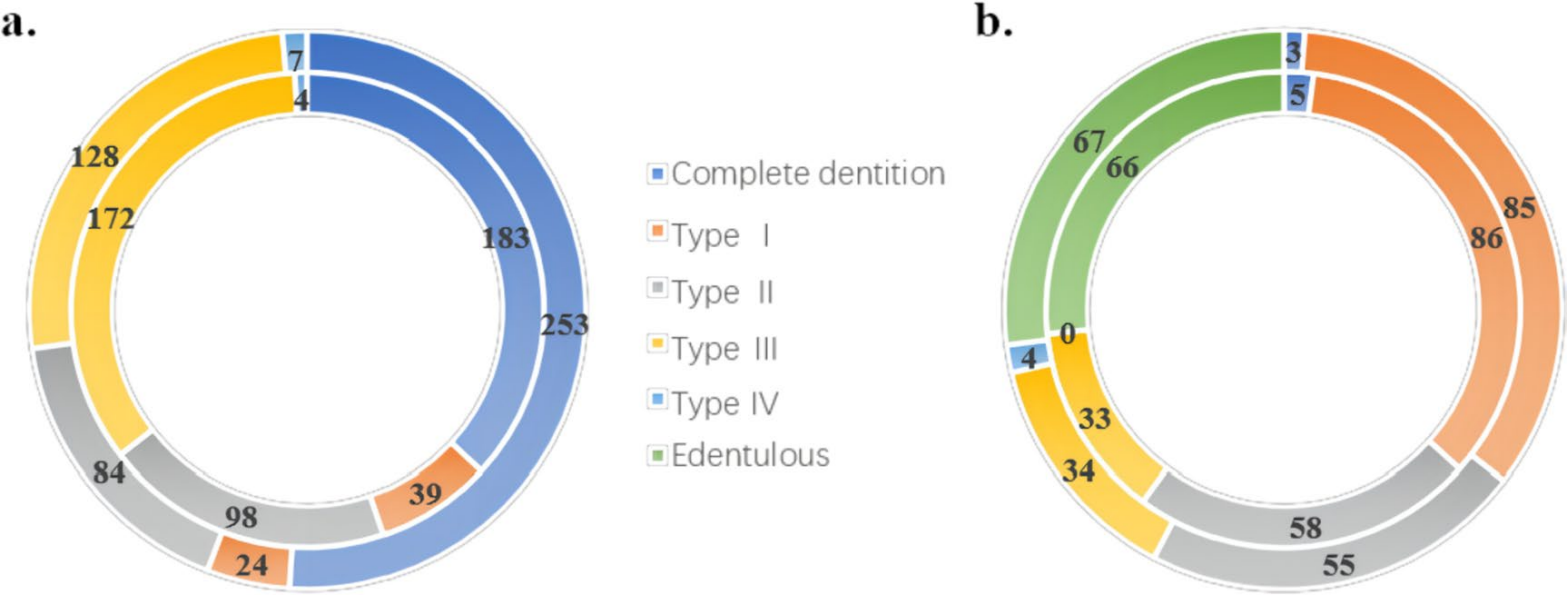
The Kennedy classification of participants (*n* = 744). Different colours indicate different classes of the Kennedy classification. The size of the rings represents the proportions of each type. The inner ring shows the classes of maxillary teeth, and the outer ring shows that of mandibular teeth. **a** the Kennedy classification of people with ≤12 functional tooth loss; **b** the Kennedy classification of people with > 12 functional tooth loss

Regarding restorations, 188 (183 of whom had lost ≤12 functional teeth) and 256 (253 of whom had lost ≤12 functional teeth) participants had no need for maxillary or mandibular restorations, respectively. Compared to those who had lost > 12 functional teeth, fewer participants were identified as Kennedy Class I (7.9% maxillary and 4.8% mandible) and Kennedy Class II (19.8 and 16.9%), but more were identified as Kennedy Class III (34.7 and 25.8%) in the group of older people who had lost fewer teeth. Sixty-six participants had a maxillary edentulous jaw, and 67 had a mandibular edentulous jaw (Fig. 3).

After adjusting for confounders, such as sex, age, educational status, level of household per capita annual income, smoking habits, drinking habits, medical history, and self-rated oral health, the number of missing functional teeth was significantly associated with the OHRQoL among older adults (OR = 1.49, 95% CI 1.05 to 2.11, *P* = .026; Table 3). However, no significant association was found between the sGOHAI score and the number of missing natural teeth (1.39, 0.93 to 2.08, *P* = .114), the number of occluding pairs (1.03, 0.99 to 1.06, *P* = .154) or functional dentition (1.43, 0.96 to 2.13, *P* = .076) after adjustment.

**Table 3:** Association between functional tooth loss, common indicators of tooth loss and the sGOHAI score (*n* = 744)

sGOHAI score	Number of functional tooth loss^**a**^		Number of natural tooth loss^**b**^		Functional dentition^**d**^		Number of occluding pairs	
	OR^**C.**^ (95% CI)	OR^**adj.#.**^ (95% CI)	OR^**C.**^ (95% CI)	OR^**adj.#.**^ (95% CI)	OR^**C.**^ (95% CI)	OR^**adj.#.**^ (95% CI)	OR^**C.**^ (95% CI)	OR^**adj.#.**^ (95% CI)
**Total sGOHAI score**	1.80 (1.32–2.45)^***^	1.49 (1.05–2.11)^*^	1.56 (1.09–2.24)^*^	1.39 (0.93–2.08)	1.58 (1.11–2.24)^*^	1.43 (0.96–2.13)	1.05 (1.02–1.08)^**^	1.03 (0.99–1.06)
**Physical function**								
1 Limit the kind of food	2.06 (1.47–2.88)^***^	1.52 (1.05–2.19)^*^	1.90 (1.27–2.85)^**^	1.50 (0.97–2.31)	1.99 (1.34–2.94)^**^	1.59 (1.04–2.43)^*^	1.08 (1.04–1.11)^***^	1.04 (1.01–1.08)^*^
2 Trouble biting/chewing	2.09 (1.50–2.90)^***^	1.58 (1.09–2.28)^*^	1.70 (1.16–2.51)^**^	1.36 (0.89–2.09)	1.74 (1.19–2.53)^**^	1.40 (0.92–2.13)	1.10 (1.06–1.13)^***^	1.07 (1.03–1.11)^***^
3 Trouble swallowing	1.56 (1.11–2.19)^*^	1.38 (0.96–1.99)	1.88 (1.28–2.76)^**^	1.72 (1.15–2.59)^**^	1.71 (1.17–2.49)^**^	1.58 (1.06–2.36)^*^	1.05 (1.01–1.08)^*^	1.03 (1.00–1.07)
4 Unable to speak clearly	5.20 (3.51–7.69)^***^	4.82 (3.17–7.33)^***^	3.99 (2.67–5.98)^***^	3.94 (2.55–6.07)^***^	3.64 (2.44–5.42)^***^	3.50 (2.29–5.37)^***^	1.15 (1.11–1.20)^***^	1.15 (1.10–1.20)^***^
**Pain and discomfort**								
5 Discomfort when eating	1.25 (0.91–1.72)	1.09 (0.77–1.53)	1.42 (0.99–2.05)	1.36 (0.92–2.02)	1.31 (0.92–1.87)	1.26 (0.86–1.84)	1.04 (1.01–1.07)^*^	1.03 (0.99–1.06)
8 Medications for pain	1.23 (0.89–1.70)	1.00 (0.71–1.41)	0.92 (0.63–1.35)	0.77 (0.51–1.15)	1.01 (0.70–1.46)	0.85 (0.58–1.26)	1.02 (0.99–1.05)	1.00 (0.97–1.03)
12 Sensitive gums	0.51 (0.37–0.70)^***^	0.45 (0.32–0.63)^***^	0.43 (0.30–0.63)^***^	0.39 (0.26–0.58)^***^	0.46 (0.32–0.65)^***^	0.42 (0.28–0.62)^***^	0.93 (0.90–0.96)^***^	0.92 (0.89–0.95)^***^
**Psychosocial impacts**								
6 Limit contact with others	2.54 (1.72–3.74)^***^	2.52 (1.65–3.83)^***^	2.19 (1.43–3.35)^***^	2.25 (1.43–3.54)^***^	2.23 (1.47–3.39)^***^	2.32 (1.49–3.62)^***^	1.08 (1.04–1.13)^***^	1.08 (1.04–1.13)^***^
7 Unhappy with appearance	1.87 (1.37–2.55)^***^	1.69 (1.20–2.37)^**^	1.64 (1.14–2.36)^**^	1.50 (1.02–2.21)^*^	1.60 (1.12–2.28)^**^	1.48 (1.02–2.16)^*^	1.05 (1.02–1.08)^**^	1.04 (1.00–1.07)^*^
9 Worried or concerned	0.77 (0.56–1.05)	0.71 (0.51–0.98)^*^	0.74 (0.51–1.06)	0.71 (0.48–1.04)	0.83 (0.58–1.18)	0.81 (0.56–1.18)	0.97 (0.94–1.00)	0.97 (0.93–1.00)^*^
10 Nervous, self-conscious	1.96 (1.38–2.78)^***^	1.76 (1.20–2.58)^***^	1.93 (1.31–2.87)^**^	1.89 (1.23–2.89)^**^	1.91 (1.30–2.81)^**^	1.89 (1.24–2.87)^**^	1.08 (1.04–1.12)^***^	1.07 (1.03–1.12)^***^
11 Uncomfortable eating in front of others	2.42 (1.72–3.40)^***^	2.12 (1.46–3.07)^***^	3.11 (1.44–3.09)^***^	1.95 (1.29–2.95)^**^	2.06 (1.41–2.99)^***^	1.92 (1.28–2.88)^**^	1.09 (1.06–1.13)^***^	1.08 (1.04–1.12)^***^

*sGOHAI score* standardized General Oral Health Assessment Index score by the mean score, *OR*^*c.*^ crude odds ratio, *OR*^*adj.*^ adjusted odds ratio, *CI*^*c.*^ crude confidence interval, *CI*^*adj.*^ adjusted confidence interval

^a^ reference to the groups with > 12 functional teeth lost, ^b^ reference to the groups with > 12 natural teeth lost; ^d^ reference to the groups without functional dentition

^**#**^Adjusted for sex, age, education level, level of household per capita annual income, smoking habits, drinking habits, medical history, and self-rated oral health

^*^P<.05, ^**^P<.01, ^***^P<.001 (highlighted in bold)

Moreover, in further analysis, functional tooth loss was shown to be related to a greater number of GOHAI items than natural tooth loss, with a greater impact on oral physical and psychosocial function and the least impact on subjective feelings such as pain and discomfort. Functional tooth loss had the most profound impact on speech ability, as people who had lost ≤12 functional teeth had greater odds of speaking clearly (4.82, 3.17 to 7.33, *P*<.001) and had fewer limitations in having contact with others (2.52, 1.65 to 3.83; *P*<.001) than those who had lost > 12 functional teeth. Compared with natural tooth loss, the number of missing functional teeth was significantly associated with food selection (1.52, 1.05 to 2.19, *P* = .025), biting/chewing ability (1.58, 1.09 to 2.28, *P* = .015), and worry or concern (0.71, 0.51 to 0.98, *P* = .040). However, the effect of natural tooth loss (1.72, 1.15 to 2.59, *P* = .009) and functional dentition (1.58, 1.06 to 2.36, *P* = .025) on swallowing ability remained significant after adjustment, while the numbers of missing functional teeth and occluding pairs did not (Table 3).

Further analysis showed that no significant difference was found in sGOHAI scores between people who lost 13–14 (1.19, 0.60 to 2.33, *P* = .621) or 13–16 (0.90, 0.52 to 1.54, *P* = .694) functional teeth and those with ≤12 missing functional teeth. However, a significant difference was found between people who had lost > 16 functional teeth and those who had lost ≤12 (0.59, 0.40 to 0.88, *P* = .009). No significant difference was found between people with ≤12 and 13–14 missing functional teeth in terms of sGOHAI scores in all items. For the cut-off of 16, no significant association was found with most items. However, people with 13–16 missing functional teeth had greater odds of speaking clearly (0.41, 0.22 to 0.77, *P* = .006) and having worry or concern about their oral health problems (1.72, 1.02 to 2.89, *P* = .041) compared with those with ≤12 missing functional teeth. Although no significant difference was found in the total sGOHAI score between the elderly individuals who had lost 13–18 functional teeth and those who had lost ≤12 missing functional teeth (0.77, 0.49 to 1.24; *P* = .280), significant differences in more items were found between those groups than between the other groups (Table 4).

**Table 4:** Association between functional tooth loss with different cut-offs and the sGOHAI score (*n* = 744)

sGOHAI score	Number of functional tooth loss		Number of functional tooth loss		Number of functional tooth loss	
	13-14^**a**^	>14^**a**^	13-16^**a**^	> 16^**a**^	13-18^**a**^	>18^**a**^
	OR^**adj.#.**^(95% CI)	OR^**adj.#.**^(95% CI)	OR^**adj.#.**^(95% CI)	OR^**adj.#.**^(95% CI)	OR^**adj.#.**^(95% CI)	OR^**adj.#.**^(95% CI)
**Total sGOHAI score**	1.19 (0.60–2.33)	0.59 (0.40–0.86)^**^	0.90 (0.52–1.54)	0.59 (0.40–0.88)^*^	0.77 (0.48–1.24)	0.60 (0.40–0.93)^*^
**Physical function**						
1 Limit the kind of food	1.30 (0.68–2.50)	0.54 (0.36–0.82)^**^	1.01 (0.59–1.73)	0.53 (0.34–0.81)^**^	0.90 (0.55–1.45)	0.51 (0.32–0.81)^**^
2 Trouble biting/chewing	1.02 (0.52–2.01)	0.56 (0.37–0.83)^**^	0.91 (0.52–1.57)	0.53 (0.34–0.81)^**^	0.83 (0.51–1.36)	0.51 (0.32–0.81)^**^
3 Trouble swallowing	1.35 (0.62–2.94)	0.64 (0.43–0.94)^*^	0.84 (0.48–1.48)	0.68 (0.45–1.02)	0.88 (0.54–1.45)	0.63 (0.41–0.97)^*^
4 Unable to speak clearly	0.49 (0.22–1.10)	0.17 (0.11–0.27)^***^	0.41 (0.22–0.77)^**^	0.16 (0.10–0.25)^***^	0.31 (0.18–0.54)^***^	0.15 (0.09–0.25)^***^
**Pain and discomfort**						
5 Discomfort when eating	1.12 (0.58–2.19)	0.88 (0.61–1.27)	1.30 (0.75–2.24)	0.79 (0.54–1.17)	1.46 (0.90–2.36)	0.66 (0.44–1.00)^*^
8 Medications for pain	0.67 (0.36–1.27)	1.10 (0.76–1.60)	0.72 (0.43–1.20)	1.17 (0.79–1.73)	0.85 (0.54–1.34)	1.14 (0.75–1.74)
12 Sensitive gums	1.81 (0.95–3.45)	2.34 (1.62–3.39)^***^	1.35 (0.81–2.27)	2.83 (1.91–4.20)^***^	1.47 (0.94–2.32)	3.11 (2.03–4.77)^***^
**Psychosocial impacts**						
6 Limit contact with others	0.53 (0.25–1.16)	0.37 (0.24–0.58)^***^	0.59 (0.31–1.13)	0.34 (0.21–0.53)^***^	0.51 (0.29–0.88)^*^	0.33 (0.21–0.54)^***^
7 Unhappy with appearance	0.65 (0.34–1.22)	0.58 (0.40–0.83)^**^	0.69 (0.41–1.16)	0.55 (0.38–0.81)^*^	0.60 (0.38–0.94)^*^	0.59 (0.39–0.88)^*^
9 Worried or concerned	1.48 (0.78–2.79)	1.40 (0.98–2.00)	1.72 (1.02–2.89)^*^	1.30 (0.90–1.89)	1.55 (0.99–2.43)	1.32 (0.89–1.98)
10 Nervous, self-conscious	1.00 (0.46–2.16)	0.51 (0.34–0.76)^**^	0.78 (0.43–1.42)	0.50 (0.33–0.76)^**^	0.75 (0.45–1.26)	0.47 (0.30–0.73)^**^
11 Uncomfortable eating in front of others	0.63 (0.31–1.29)	0.44 (0.30–0.66)^***^	0.58 (0.33–1.02)	0.43 (0.29–0.65)^***^	0.60 (0.37–0.98)^*^	0.40 (0.26–0.61)^***^

*sGOHAI score* standardized General Oral Health Assessment Index score by the mean score, *OR*^*c.*^ crude odds ratio, *OR*^*adj.*^ adjusted odds ratio, *CI*^*c.*^ crude confidence interval, *CI*^*adj.*^ adjusted confidence interval

^a^ reference to the groups with ≤12 functional teeth lost

^**#**^Adjusted for sex, age, education level, level of household per capita annual income, smoking habits, drinking habits, medical history, and self-rated oral health

^*^P<.05, ^**^P<.01, ^***^P<.001 (highlighted in bold)

Further subgroup analyses showed significant associations between losing the second premolars (0.60, 95% CI 0.41 to 0.87, *P* = .012), first molars (0.65, 0.45 to 0.93, *P* = .018), or second molars (0.58, 0.40 to 0.83, *P* = .003) and the sGOHAI score when people had lost ≤12 functional teeth. For those who had lost ≤16 functional teeth, similar associations with the sGOHAI score were also found for the second molars (0.60, 0.42 to 0.84, *P* = .003), first molars (0.64, 0.46 to 0.90, *P* = .009) and second premolars (0.62, 0.44 to 0.87, *P* = .006). Moreover, among people who had lost > 16 functional teeth, a significant association was observed between the loss of central incisors and the sGOHAI score (8.78, 1.11 to 69.60, *P* = .015), while no such relationship was detected in other groups (Table 5).

**Table 5:** Stratified chi-square test of positions of tooth loss and sGOHAI score in the subgroups (*n* = 744)

Subgroups of functional tooth loss		Total sGOHAI score		
		Pearson χ^**2**^ value	OR	95% CI
When the cut-off was 12	≤**12**			
	Lost second molars	8.81^**^	0.58^**^	0.40, 0.83^**^
	Lost first molars	5.68^*^	0.65^*^	0.45, 0.93^*^
	Lost second premolars	6.65^*^	0.60^*^	0.41, 0.87^*^
	Lost first premolars	0.33	0.88	0.56, 1.37
	Lost canine teeth	1.41	1.62	0.73, 3.62
	Lost lateral incisor	0.03	1.04	0.63, 1.72
	Lost central incisor	0.00	0.99	0.60, 1.64
	> **12**			
	Lost second molars	0.06	1.24	0.20, 7.56
	Lost first molars	0.02	0.82	0.05, 13.30
	Lost second premolars	0.01	0.96	0.31, 2.94
	Lost first premolars	0.85	0.62	0.22, 1.72
	Lost canine teeth	2.03	0.67	0.39, 1.16
	Lost lateral incisor	2.93	0.56	0.29, 1.09
	Lost central incisor	0.04	0.94	0.52, 1.73
When the cut-off was 16	≤**16**			
	Lost second molars	8.57^**^	0.60^**^	0.42, 0.84^**^
	Lost first molars	6.75^**^	0.64^**^	0.46, 0.90^**^
	Lost second premolars	7.60^**^	0.62^**^	0.44, 0.87^**^
	Lost first premolars	1.11	0.82	0.57. 1.19
	Lost canine teeth	0.01	0.98	0.56, 1.71
	Lost lateral incisor	0.82	0.83	0.55, 1.25
	Lost central incisor	0.56	0.85	0.56, 1.30
	> **16**			
	Lost second molars	1.40	1.01	0.99, 1.04
	Lost first molars	-^a^		
	Lost second premolars	1.46	0.98	0.95, 1.01
	Lost first premolars	0.77	0.35	0.03, 3.98
	Lost canine teeth	< 0.01	1.01	0.42, 2.42
	Lost lateral incisor	0.07	1.21	0.28, 5.24
	Lost central incisor^b^	5.96^*^	8.78^*^	1.11, 69.60^*^

*OR* odds ratio, *CI* confidence interval

^a^ Failed to report the result because the variable was constant

^b^ The results were unstable due to the limited sample size and skewed data distribution

^*^P<.05, ^**^P<.01, ^***^P<.001 (highlighted in bold)

## Discussion

The present cross-sectional analysis of 744 older individuals aged 65–74 years comprehensively explored the relationship between tooth loss and OHRQoL. Compared with natural tooth loss, functional dentition, or occluding pairs, a more positive relationship was found between losing ≤12 (even ≤14 or ≤ 16) functional teeth and a better OHRQoL, especially in the physical and psychosocial function domains; the hypothesis of our study was confirmed. These findings highlight the importance of maintaining both tooth number and tooth function in the older population and establishing a new measure for good OHRQoL and successful oral ageing. The results were relatively reliable and showed good extrapolation and generalizability, with representative samples and effective control of the relevant bias.

Unlike the previous controversial results reported for common indicators [13, 32, 38, 39], the current study proposed the concept of functional tooth loss and explored its significant association with the OHRQoL of older persons, which mostly manifested in oral physiological and psychosocial functions. It was shown that functional tooth loss had greater effects on the OHRQoL of older adults than other common indicators, such as natural tooth loss, functional dentition, and occluding pairs. With increasing functional tooth loss, difficulties with food limitation, chewing difficulty, and speaking trouble were aggravated, which was consistent with previous studies [18, 20, 31]. Difficulties with smiling, speaking, aesthetics, and the social aspects of food brought by functional tooth loss might result in impaired self-esteem and social status as well as reduced emotional stability, thereby frustrating interpersonal relationships and leading to social fears and disorders [7, 38–40]. However, the effect of functional tooth loss on oral pain and discomfort was not detected in our study. The theory of response shift could be used to explain the irrelevance between tooth loss and oral pain. As a result of timing and the experience of poor health, older people might change their internal standards, values, or conceptualization of OHRQoL, which makes oral discomfort and pain unimportant at this point in their lives [1, 41].

However, compared with functional tooth loss, natural tooth loss and functional dentition were found to be significantly associated with swallowing function. We supposed that as a result of nonfunctional roots and third molars without chewing force, soft and chewable food was more likely to be subjectively chosen by the older population [20, 39, 42], which also contributed to the decrease in swallowing problems of older people [21, 42] and the increase in psychological stress [31]. When compared with occluding pairs, the relationship between functional tooth loss and physical or psychosocial function was not inferior, and it even had a more significant effect on the overall OHRQoL of older people. Moreover, without the need to record the occlusal relationship in detail, functional tooth loss might be more suitable for large-scale epidemiological investigation, which could be more convenient for quick inspection or recording.

Interestingly, we found that a smaller number of functional teeth was sufficient to maintain oral function in older participants. The older adults with 18–19 functional teeth (equal to the loss of 13–14 functional teeth) had a similar OHRQoL compared with those with ≥20 functional teeth remaining (i.e., the minimum number of teeth needed to maintain OHRQoL, according to the previous consensus). Moreover, compared with those who had lost ≤12 functional teeth, people with 13–16 missing functional teeth showed more difficulty speaking clearly and were more likely to be worried and concerned about their oral health problems. The results might be related to the significant association between central incisors and OHRQoL in the subgroup analysis, as anterior teeth, especially central incisors, generally have an important role in aesthetics and speech. However, more research with a larger sample size is needed to confirm this finding due to the limited sample size and skewed data distribution in the subgroup analysis. Even so, considering that these differences seemed to be acceptable while evaluating the OHRQoL of older people, we proposed that 16 functional teeth seemed to be the minimal requirement for maintaining good OHRQoL and successful oral ageing, which emphasized the importance of maintaining the function of remaining teeth rather than preserving or increasing the number of teeth in oral medicine.

Therefore, considering the importance of maintaining oral function via health promotion and disease prevention, several public health implications should be highlighted. On the one hand, a prevention-oriented oral medical system should be established to reduce the impact of functional tooth loss on OHRQoL and the economic burden of the older population, as tooth loss and the consequent oral diseases are preventable by promoting regular oral health care [11, 16, 43]. On the other hand, the goal of maintaining oral function should be prioritized in each clinical decision to ensure the acceptable OHRQoL of patients [13, 31]. Clinicians should make a comprehensive assessment of the impacts of any treatment modality on patients’ quality of life to provide patient-centred oral health care [13].

However, there were several limitations in this study. First, relevant data on functional tooth loss, natural tooth loss, functional dentition, and occluding pairs were obtained from the original records, which might introduce some bias to the study. Prospective studies evaluating occlusal functional or psychosocial function are needed to explore the differences in these indicators in clinical applications and to validate the results in the future. Second, the contribution of removable dentures to OHRQoL, of which the impact on oral function was reported to be controversial [31], was not involved in this study. The contribution of removable dentures to OHRQoL is reported to be difficult to evaluate in the short term due to the adaptation curve [24] and is inconclusive in the long term because of biological complications, including but not limited to caries and periodontal diseases [44]. Studies have shown that the function of removable dentures differs based on the position of replacement, denture self-satisfaction, patient outcome expectations and so on [33, 34]. Relevant research is needed to determine the effects of removable dentures on OHRQoL. Moreover, more experiments should be conducted to explore the mechanism of the impact of functional tooth loss on OHRQoL; for example, objective indicators are needed to evaluate changes in chewing function, swallowing function and other oral functions.

## Conclusion

Functional tooth loss can be a better indicator of OHRQoL in the older population than some common indicators, such as natural tooth loss, functional dentition and occluding pairs. As the number of missing functional teeth increases, the OHRQoL of older people significantly decreases, particularly in the domains of physical and psychosocial functions. Moreover, at least 16 functional teeth seem to be the new measure of good OHRQoL and successful oral ageing. Global strategies aimed at preventing tooth loss and maintaining oral functions are needed to maintain acceptable OHRQoL in the ageing population.

## Data Availability

The datasets used and analysed during the current study are available from the corresponding author upon reasonable request.

## Abbreviations

OHRQoL: Oral health-related quality of life
GOHAI: The 12-item Geriatric Oral Health Assessment Index
sGOHAI: Standardized Geriatric Oral Health Assessment Index
WHO: World Health Organization
STROBE: Strengthening the Reporting of Observational Studies in Epidemiology
PPS: Probability proportional to size
SD: Standard deviation
OR: Odds ratio
CI: Confidence interval
